# The Epigenetic Horizon: New Molecular Perspectives

**DOI:** 10.3390/ijms27104541

**Published:** 2026-05-19

**Authors:** Marco Miceli

**Affiliations:** 1 CEINGE-Biotecnologie Avanzate Franco Salvatore, 80145 Naples, Italy; micelim@ceinge.unina.it; 2 Department of Neuroscience, Reproductive Sciences and Dentistry, University of Naples Federico II, 80131 Naples, Italy

The term “epigenetics” comes from the union of the words “epigenesis” and “genetics”, coined by the British embryologist Conrad Waddington in 1942 [1], to contrast the preformationist theory, prevalent at that time, according to which adult organisms are already preformed in miniature (germini) in the egg or in the sperm, but develop through continuous processes from a single cell (fertilized oocyte) [2], underlining how embryonic development and cellular differentiation are the result of a complex interaction between the genetic makeup and the influences of the environment.

The first epigenetic modification to be brought to light, between the 60 s and 70 s, was the methylation of the nitrogenous bases of DNA (known since the 40 s) [3]. Since then, new and interesting epigenetic mechanisms have been discovered, among which, in addition to the already mentioned methylation, hydroxymethylation (5-hydroxymethylcytosine, 5hmC) stands out, with modifications that occur directly on the nitrogenous bases of DNA, influencing the accessibility of the transcriptional machinery to the gene promoter; post-translational modifications of histones (PTMs) such as acetylation, methylation, phosphorylation, ubiquitination, sumoylation, lactylation, etc., “molecular switches” that modify the structure of chromatin (open/accessible or closed/compacted) and determine whether a gene is turned on (expressed) or turned off (silenced); mechanisms mediated by non-coding RNAs such as microRNAs (miRNAs) and Long non-coding RNAs (lncRNAs) that act at the transcriptional and post-transcriptional levels, modulating gene expression, splicing and stability [4,5,6].

More recently, research has identified a wide range of less common modifications that greatly expand the complexity of the cellular “language”, such as crotonylation, acetylation-like butyrylation, and propionylation [7]. Glutanylation and ADP-ribosylation [8], modifications that play key roles in the response to oxidative stress and in DNA damage repair. Serotonylation [9] and dopaminylation [10] directly link to histones (especially H3), influencing gene expression in neurons, etc.

In this context, the Special Issue “Molecular Research on Epigenetic Modifications” aims to provide a significant scientific platform for furthering our understanding of epigenetic mechanisms through a carefully selected series of three original articles and three reviews. The focus of these articles is not only to describe the underlying molecular processes but also to understand their crucial impact on human health, cellular development, and responses to environmental stimuli, whether arising from chemical/physical stressors or from microorganisms such as viruses.

In this scenario, epigenetics becomes a keystone for understanding biological plasticity, paving the way for new and innovative therapeutic strategies. It encompasses diverse topics, from the study of circulating cell-free DNA as an early diagnostic tool, to the interaction between the gut microbiota, epigenetic mechanisms, and metabolic health, to the study of classical demethylases, epigenetic mechanisms in viruses and eukaryotic cells, and autoimmune diseases. Let us examine a short excerpt from each of these topics.

In the study by Qvick et al. [11], differentially methylated regions (DMRs) in circulating cell-free DNA (cfDNA) are analyzed as an early, novel diagnostic tool. The clear methylation differences and the DMR-based classification support cfDNA methylation as a robust biomarker for cancer detection in patients with confounding conditions, highlighting how analyzing the epigenetic profile of cfDNA overcomes the limitations of classical protein biomarkers, which are often influenced by inflammatory states. By identifying specific DMRs, researchers were able to precisely distinguish and isolate unique molecular “signatures” even in the presence of chronic diseases or confounding conditions that normally generate false positives.

The study by Agodi et al. [12] analyzes the impact of bariatric surgery on non-alcoholic fatty liver disease (NAFLD), exploring the complex interaction between gut microbiota, epigenetic mechanisms, and metabolic health.

Through DNA methylation analysis, the researchers identified significant changes in CpG sites between baseline and six months post-surgery. These changes predominantly affect genes involved in the autophagy pathway, suggesting a restoration of cellular degradation processes essential for the resolution of liver damage.

The results confirm that responses to surgery are highly individualized and highlight the importance of integrating epigenetic profiling and microbiota monitoring into clinical management. This approach paves the way for personalized therapeutic strategies capable of optimizing clinical outcomes and improving the prognosis of patients with NAFLD.

The work of Cruz et al. [13] demonstrates that the demethylase JMJD1B is essential for genome stability, as it ensures the proper influx of histones H3 and H4 into the nucleus. Its deficiency hinders chromatin assembly, causing DNA damage, while its presence in melanoma drastically reduces the accumulation of oncogenic mutations, acting as a safeguard against genomic instability.

The review by Cevenini et al. [14] offers a detailed analysis of how human cytomegalovirus (HCMV) manages to persist in the host for entire decades, alternating periods of absolute silence (latency phase) with sudden phases of active replication (lytic phase), in which the key element of this biological “switch” is epigenetic regulation. The process begins almost instantaneously: just 30 minutes after entering the cell, the viral DNA (which enters as a naked molecule) begins to organize itself into nucleosomes, acquiring a chromatin structure similar to that of the human genome.

The review by Schiano et al. [15] analyzes how the integrity of the vascular system is compromised by shared molecular mechanisms, with a focus on extracellular vesicles (EVs) as mediators of vascular remodeling and neoangiogenesis. The authors highlight the role of exosomes as reversible epigenetic switches, capable of triggering functional modifications of the vessel in both cardiovascular diseases and cancer.

The review by Pesqueda-Cendejas et al. [16] highlights the role of epigenetic alterations, particularly DNA hypomethylation, in the pathogenesis of Systemic Lupus Erythematosus (SLE). These changes can be influenced by diet, making nutrition a potential adjuvant therapeutic tool.

The future of epigenetics. Born as a branch of modern genetics and considered one of the most important revolutions in biology in recent decades, it has yet to fully live up to expectations regarding its enormous intrinsic potential.

The extreme complexity that arises from the interaction between DNA and the environment through countless reversible modifications, compared to classical genetics, has contributed to the stratification of complex, difficult-to-understand superstructures that, once revealed, will lead to unparalleled scientific/technological development.

The “near” future of medicine lies in this ability to decode and modulate interactions between the genome and environmental stimuli, transforming genetic susceptibility into a manageable and reversible parameter.

The main areas of development can be summarized in 5 key points: (i) Personalized Medicine and Longevity: The creation of epigenetic maps of aging allows to determine the biological age of individual organs, paving the way for targeted interventions to slow down cellular decline [17]; (ii) Oncology and Early Diagnostics: The use of “machine learning” to analyze epigenetic markers is improving the early diagnosis of complex tumors, such as juvenile osteosarcoma [18]; (iii) Neuroscience and Psychiatry: Interventions are being studied to modulate the epigenetic response in diseases such as Alzheimer’s, depression and schizophrenia, influencing processes such as neuroinflammation, etc. [19]; (iv) Nutrigenetics and Sport: The integration of epigenetic data will allow to optimize athletic performance and health through diets and lifestyles “designed” on the individual epigenetic profile [20]; (v) Transgenerational Inheritance: Research is investigating how parents’ traumas and environmental exposures can be transmitted to future generations, offering new insights into family health prevention [21].

## Data Availability

No new data were generated for this editorial. The citations in this article have been reworked from scientific articles as indicated in the bibliography.
